# Passed the Age of Puberty: Organizational Networks as a Way to Get Things Done in the Health Field: Comment on "Evaluating Global Health Partnerships: A Case Study of a Gavi HPV Vaccine Application Process in Uganda"

**DOI:** 10.15171/ijhpm.2017.51

**Published:** 2017-05-03

**Authors:** Patrick Kenis

**Affiliations:** ^1^Tilburg University, Tilburg, The Netherlands.; ^2^WU Vienna University of Economics and Business, Vienna, Austria.

**Keywords:** Partnership, Organization Network, Social Network Analysis, Evaluation, HPV Vaccine Coverage

## Abstract

In this commentary I will demonstrate that the case study of Uganda’s Human papilloma virus (HPV) vaccine application partnership provides an excellent example of widening our lens by evaluating the successful HPV vaccine coverage from a network-centric perspective. That implies that the organizational network is seen as the locus of production and that network theories become indispensable to analyze the situation at hand. The case study is, as said, an excellent example of how this can be done and my comments have to be read as an endorsement and a broadening of the discussion of what Carol Kamya and colleagues have presented. It is demonstrated that an organizational network approach can be considered a serious and mature way in understanding public health issues.

## Introduction

The study of Uganda’s Human papilloma virus (HPV) vaccine application partnership provides an excellent opportunity to illustrate the strength and challenges in working with a partnership approach in the health field (and beyond). ‘Partnership’ is, however, a rather vague and hardly discriminating concept and one could even ask ‘who could be against partnerships?’ and thus, ‘why care?’ I would like to illustrate in the following that we should care and that partnerships do matter but only if we are specific and clear about our level of analysis. The gist of my commentary is that the authors are very successful with their analysis because they moved beyond ‘partnership’ towards a network-centric level of analysis.

I would like to take this opportunity to elaborate on the insights of this study in order to point to a number of broader issues which were probably beyond the (page) limit of this publication and which deserve attention and can inform our future thinking about the functioning of what the authors call partnerships. I do this by presenting six key ideas for understanding the type of partnership presented in the study. I will not repeat what can be read in the article but the six key ideas should certainly be read as an endorsement for the importance of the study by Carol Kamya and colleagues.^1^

### First: the study recognizes ‘partnerships’ as a unique way of getting things done.

What the study clearly illustrates is that partnership is a way to get things done (in their case, the provision of HPV vaccine immunization). It becomes clear that partnership is a tool and not an end in itself (contrary to the UN Sustainable Development Goals, where developing partnership is considered a goal in itself). This is important because it obliges us to think about when this tool is appropriate and when it is not. Common organizational study perspectives (such as transaction-cost theory) would advise that if an organization can achieve a task on its own, it should do it alone. The reason is that a single organization can follow its own strategy, control its own resources and evaluate how it is being done. Collaborating with others, on the other hand, involves (often underestimated) transaction and coordination costs and therefore, an organization is regarded to be better off doing it alone. But, what if a single organization does not have sufficient capabilities for achieving the goal and/or the issue at hand is so complex that we cannot even conceive of a single (even newly established) organization to do the job? In such a case it becomes clear that partnerships are imperative. But, it needs to be well justified why a partnership is essential as done in the paper by Carol Kamya and colleagues.^1^ Personally, I prefer the word “organizational network” over “partnership” since it better points us to the fact that a deliberate and goal-directed multi-actor setting has been considered for the task at hand. The term partnership, on the other hand, communicates the connotation of a somewhat voluntarily, personalized and bi-lateral (instead of multi-lateral) type of setting. It underrates in some way the uniqueness of what the deliberate and goal-directed multi-actor system achieved in practice. The case of the “Uganda’s HPV vaccine application partnership” is convincingly presented as a case where a set of diverse actors from different organizations need to work towards a common goal (in the absence of a clear formal authority or ownership of the problem) and build, maintain and evaluate this type of organizational design.

### Second: the study recognizes the partnership configuration as the locus of production.

Based on the previous point it is important to note that the study recognizes the fact that the locus of production for a successful HPV vaccine coverage is the organizational network and thus uses a network-centric approach. This might sound obvious but moving from the organization to network as the locus of production is often a big step in practice and for theory. This argument has convincingly been put forward by Mandell and colleagues^2^ in their publication entitled “Collaborative networks and the need for a new management language.” In their paper they argue that theory and practice “remain largely focused on the narrow transactions between organizations in the network to secure scare resources.” They further criticize an emphasis on ‘the need for management strategies that protect boundaries, buffer dependency and treat relationships as resources in order to gain competitive advantage” rather than studying and focusing on “the processes that occur within these types of networks – especially those that relate to the formation of a cohesive unit (or whole) which is one of the key characteristics of an effective collaborative network.” Again, the paper is an excellent example in which the authors have studied the HPV vaccine application as the result of an integrated system and moved on to identifying appropriate tools to study the whole rather than merely focusing on the individual parts of the system.

### Third: The study shows how important it is to move beyond a general public health discourse to point to the importance of achieving a concrete and valuable outcome.

The study not only moves beyond a focus on the individual parts but also avoids to move into a general (and often superficial) public health policy discourse in which partnerships and collaboration becomes a kind of doctrine. The article distinguishes implicitly, although in different terms, between the ‘network governance’ or ‘collaborative governance’ and the ‘governance of networks’ or ‘governing collaboration’ (for this argument see also Siv Vangen, et al^3^). We can easily agree that in many cases next to the more commonly known market mechanisms, hierarchical mechanism or bi-lateral collaborative mechanisms we need an organizational network approach to get things done. Too often, however, studies leave it at this and do not move to the more pertinent question why some of these networks function better than others and how they can be governed effectively (ie, addressing the ‘governance of networks’). Promoting ‘network governance’ as a new kid on the block is fairly easy these days given the positive attention it gets, but moving further and deepening into the practice and study of the ‘governance of organizational networks’ is another ballgame. This ballgame is taken up by the paper on the Uganda’s HPV vaccine application partnership by introducing two instruments: the partnership framework and network mapping to better understand how an organizational network functions and what exactly holds it together (in the absence of formal authority and ownership). The paper is exemplary in this.

### Fourth: It recognizes the multi-complexity of the situation.

Governance of organizational networks would be fairly straightforward in case the network is not much diversified and/or when it would be clear who is in charge. The article clearly points out that this is hardly the case and that the complex situations need to be approached in a complex way. This is in line with one of the two megatrends put forward in the recently published report “New Directions in Governing the Global Health Domain” (Kickbusch et al^4^): “The dominant approach to govern the global health domain is increasingly through building and shaping cross-sectoral networks, creating hybrid organisations and enabling dynamic policy alignments, which work to voluntary rules.” This new reality (which has also been described in Provan and Kenis,^5^ and Raab and Kenis^6^) is clearly recognized by the authors of the article. It has led them to look for concepts and instruments for their analysis and to contribute implications for policy makers in dealing with this new reality.

### Fifth: It recognizes the need to make the connections in organizational networks visible in order to better understand whether the structure contributes (or not) to meet the expected outcome.

In the absence of clear lines of authority and responding to the observation that “everything is connected to everything else: but how?”^4^ we need instruments to make these connections visible. The article presents a network analysis and is thus able to point to the degree of comprehensiveness, integration and collaboration within the organizational network and thus can address the question posed by Kickbusch et al.^4^ It clearly demonstrates the fact that information obtained through network analysis can be used to analyze and help to build capacity through the development of a stronger network of collaborating organizations (see also Provan et al^7^). It should be applauded that the authors used network analysis tools to make the connections in the organization network visible. Such a structural analysis proves helpful but it would have been carried the analysis even further if it had been combined with a process-oriented analysis as structures evolve as parties interact over time.^8^ Such a combined analysis could be the key to understand the structure and dynamics in public health issues because such a process-oriented approach takes into account the nonlinear and emergent nature of collaboration over time.

### Sixth: It demonstrates that also partnerships or organizational networks can be evaluated.

We increasingly observe organizational networks as a promising way to get things done and we are developing new approaches and a new language for describing and analyzing them as we have seen above. But, if we are not able to demonstrate to what extent they meet the complex challenges they are designed to address we are missing something. This might seem too obvious to mention but if we seriously take organizational networks as the locus of production this becomes very challenging. Market governance (“are my products sold or not”), hierarchical governance (“is my superior happy with my performance or not”) and bilateral partnerships (“are we still happy being together”) can be evaluated in a rather straightforward way. The case of organizational networks is quite different. We could, of course, assess an organizational network on its outcome (eg, degree of successful HPV vaccine coverage). The problem, however, is that studies have shown that organizational networks might need several years to produce their added value (see Raab et al^9^). Before producing network-level results investments are needed to build the network. For example, it takes time for participating organizations to break down their boundaries and to realize that they have become part of a new entity. In addition, they have to realize that they now create something jointly that is considered a purposeful common need by others. Rather than being paralyzed by this complexity in studying the effectiveness of organizational networks, the authors bravely took up the gauntlet. They have done so by presenting and applying a partnership analysis framework. The framework produces important insights and points to “key drivers of partnership added value.” How applicable and significant the framework is to other situations cannot be answered here. The framework is certainly a promising step as a starting point to be used in a prospective evaluation and thus help to further develop the framework. The article by Carol Kamya and colleagues^1^ is a great source to see how this can be done.

## Conclusion

It can be concluded that the paper by Carol Kamya and colleagues demonstrates that the usage of an organizational network approach in the health field has passed the age of puberty. How this approach contributes to another and probably improved understanding of public health issues has been argued in the different points presented above: public health issues are best analyzed at the network-level of analysis, if achieving a goal exceeds the capacity of the single organization. The complexity of public health issues should be recognized and analyzed with appropriate complex tools and they need the ambition to demonstrate, at the end of the day, whether they produce explainable outcomes. All these led me to argue against the word ‘partnerships’ in such contexts. Partnership might sound more sympathetic but it should be clear that engaging with organizational networks is much more than an acte gratuit and needs building, sustaining and evaluating. This is exactly what the study nicely demonstrates.

## Ethical issues

Not applicable.

## Competing interests

Author declares that he has no competing interests.

## Author’s contribution

PK is the single author of the paper.
